# Supplementary material for: Community-based health insurance beneficiaries’ satisfaction on laboratory services and associated factors in selected public hospitals in Jimma Zone, Oromia Region, Southwest, Ethiopia
Source: PLoS One. 2024 Aug 14;19(8):e0308814. doi: 10.1371/journal.pone.0308814 (PMC11324112; doi:10.1371/journal.pone.0308814)
Supplement: S1 File — (DOCX) [file pone.0308814.s001.docx]

Total CBHI beneficiaries in study Hospitals

**88,449**

Agaro General Hospital

36,652

Shenen Gibe General Hospital

12,285

Seka Chokorsa Hospital

39,512

174

59

188

Proportional allocation to each hospital

**Final selected participants 421**

Convenient sampling technique

Sampling procedures used to reach study subjects.

**JIMMA UNIVERSITY**

**INSTITUTE OF HEALTH**

Consent Forms

Participant Code Number__________

Study participants will be informed about required information. I am informed fully in the language I understand about the aim of the above-mentioned research. I understood the purpose of the study entitled with ‘To assess level of community-based health insurance beneficiaries’ satisfaction on medical laboratory services and its associated factors among selected public hospitals in Jimma zone, south west Ethiopia’

I have been informed that, the data will be gathered from me and there will be minimal risk during data collection. In addition, I have been told all the information collected throughout the research process will be kept confidential. The secrecy of information will be secured by anonymity. I have the right to withdraw myself from the research at any time without being affecting about my medical care. I have given my consent freely to participate in the study. Agree ________ Not agree________

Therefore, I gave my consent without any coercion for my participation in this study.

Participant’s Signature ______ Date___________

Data collector’s name _________________ Signature _______ Date__________

Witnesses: 1. Name _________________ Signature _______ Date _______

2. Name _________________ Signature _______ Date________

**1. Socio-Economic Demographic Data**

Questionnaires: To assess level of community-based health insurance beneficiaries’ satisfaction on medical laboratory services and its associated factors among selected public hospitals in Jimma zone, south west Ethiopia

1. Participant code: _________________

| S. N. | Questions & filter | Coding category |
| --- | --- | --- |
| 101 | Age | 1) 18-24 2) 25-34 3) 35-44 4) 45-54 5) >55 |
| 102 | Gender | 1) Male 2) Female |
| 103 | Language | 1) Afaan Oromo 2) Amharigna 3) other language |
| 104 | Marital status | 1) Single 2) Married 3) Divorced/widowed |
| 105 | Household family size | 1) 1-5 2) 6-10 3) 11-15 4) >16 |
| 106 | Educational level | 1) Illiterate 2) Read and write 3) Elementary education |
| 107 | Occupation | 1) Farmer 2) Merchant 3) Daily laborer 4) Other |
| 108 | Number of family size | 1) 1-5 2) 6-10 3) 11-15 4) above 16 |
| 109 | Household wealth index /family income (self-report) | 1) 100USD 2) 200USD 3) 300USD 4) 400USD |
| 110 | Residence | 1) Rural 2) urban |
| 111 | Number of hospital visits | 1)1 2) 2 3)3 4) 4 5) >5 |

1. **Laboratory services provision-related determinants of CBHI members’ satisfaction**

| 201 | The availability of the requested laboratory tests | 1. Strongly Dissatisfied 2. Dissatisfied  3. Neutral 4. Satisfied 5.Strongly Satisfied |
| --- | --- | --- |
| 202 | Availability of proper and clear direction of each lab rooms/sections | 1. Strongly Dissatisfied 2. Dissatisfied  3. Neutral 4. Satisfied 5.Strongly Satisfied |
| 203 | Services providers friendly how they well come to you? | 1. Strongly Dissatisfied 2. Dissatisfied  3. Neutral 4. Satisfied 5.Strongly Satisfied |
| 204 | Are you happy with laboratory opening time? | 1. Strongly Dissatisfied 2. Dissatisfied  3. Neutral 4. Satisfied 5.Strongly Satisfied |
| 205 | Availability of sufficient reception area | 1. Strongly Dissatisfied 2. Dissatisfied  3. Neutral 4. Satisfied 5.Strongly Satisfied |
| 206 | Availability of sufficient information provision | 1. Strongly Dissatisfied 2. Dissatisfied  3. Neutral 4. Satisfied 5.Strongly Satisfied |
| 207 | Availability of entertaining materials at the lab result waiting area | 1. Strongly Dissatisfied 2. Dissatisfied  3. Neutral 4. Satisfied 5.Strongly Satisfied |
| 208 | Procedures for blood and other body fluid specimen collection | 1. Strongly Dissatisfied 2. Dissatisfied  3. Neutral 4. Satisfied 5.Strongly Satisfied |
| 209 | Duration of waiting time for lab test result collection | 1. Strongly Dissatisfied 2. Dissatisfied  3. Neutral 4. Satisfied 5.Strongly Satisfied |
| 210 | Availability of benches or chairs sitting arrangements in waiting area according to | 1. Strongly Dissatisfied 2. Dissatisfied  3. Neutral 4. Satisfied 5.Strongly Satisfied |
| 211 | Covid-19 prevention and controlling protocol standards | 1. Strongly Dissatisfied 2. Dissatisfied  3. Neutral 4. Satisfied 5.Strongly Satisfied |
| 212 | Maintenance of patients’ privacy and confidentiality in laboratory rooms | 1. Strongly Dissatisfied 2. Dissatisfied  3. Neutral 4. Satisfied 5.Strongly Satisfied |
| 213 | Comfortable with the laboratory setup or organizational structure | 1. Strongly Dissatisfied 2. Dissatisfied  3. Neutral 4. Satisfied 5.Strongly Satisfied |
| 214 | Cleanliness of the laboratory rooms | 1. Strongly Dissatisfied 2. Dissatisfied  3. Neutral 4. Satisfied 5.Strongly Satisfied |
| 215 | The availability of the requested laboratory tests | 1. Strongly Dissatisfied 2. Dissatisfied  3. Neutral 4. Satisfied 5.Strongly Satisfied |
| 216 | Duration of waiting time to get laboratory test results | 1. Strongly Dissatisfied 2. Dissatisfied  3. Neutral 4. Satisfied 5.Strongly Satisfied |
| 217 | The location/clear direction of the laboratory to patients | 1. Strongly Dissatisfied 2. Dissatisfied  3. Neutral 4. Satisfied 5.Strongly Satisfied |
| 218 | Cleanness and comfort of the latrine | 1. Strongly Dissatisfied 2. Dissatisfied  3. Neutral 4. Satisfied 5.Strongly Satisfied |
| 219 | The ability of service provider explanation about diagnostic test during sample collection | 1. Strongly Dissatisfied 2. Dissatisfied  3. Neutral 4. Satisfied 5.Strongly Satisfied |
| 220 | Availability of laboratory staff on working hours | 1. Strongly Dissatisfied 2. Dissatisfied  3. Neutral 4. Satisfied 5.Strongly Satisfied |
| 221 | Laboratory personnel’s professional appearances (neatness, professional dressing) | 1. Strongly Dissatisfied 2. Dissatisfied  3. Neutral 4. Satisfied 5.Strongly Satisfied |
| 222 | Duration of CBHI users ID receiving time | 1. Strongly Dissatisfied 2. Dissatisfied  3. Neutral 4. Satisfied 5.Strongly Satisfied |
| 223 | Laboratory service continuity | 1. Strongly Dissatisfied 2. Dissatisfied  3. Neutral 4. Satisfied 5.Strongly Satisfied |
| 224 | Distance from nearby health care facility | 1. Strongly Dissatisfied 2. Dissatisfied  3. Neutral 4. Satisfied 5.Strongly Satisfied |
| 225 | Patients with chronic diseases rate of satisfaction | 1. Strongly Dissatisfied 2. Dissatisfied  3. Neutral 4. Satisfied 5.Strongly Satisfied |
| 226 | What challenge did you faced while getting laboratory service other than the above-mentioned issues? | 1. Strongly Dissatisfied 2. Dissatisfied  3. Neutral 4. Satisfied 5.Strongly Satisfied |

Thanks for have time with you!
