# Supplementary material for: Community-based health insurance beneficiaries’ satisfaction on laboratory services and associated factors in selected public hospitals in Jimma Zone, Oromia Region, Southwest, Ethiopia
Source: PLoS One. 2024 Aug 14;19(8):e0308814. doi: 10.1371/journal.pone.0308814 (PMC11324112; doi:10.1371/journal.pone.0308814)
Supplement: Attachment — Submitted filename: Response to Reviewers.docx [file pone.0308814.s004.docx]

**Point-by-point response to review questions**

Dear Editor,

Thank you for facilitating the review of our manuscript and the reviewers for their invaluable comments. After careful review of all the invaluable comments raised by the editor and reviewers, we have prepared a point-by-point response and also revised the manuscript accordingly.

Additional Editor Comments:

Reviewer #1:

I person I am satisfied with the responses and want to give credit for the great work. I would only like to recommend the authors recheck the reference format, e.g., 4, 7, & 8.

**Response**: thank you very much for this important concern. I have corrected it in the revised copy of the article.

Reviewer #2:

Most of the comments have been addressed, but I would recommend you either rephrase or revise the eligibility criteria

**Response**: thank you very much for this important concern. I have corrected it in the revised copy of the article.
